# A case of recurrent giant cell tumor of bone with malignant transformation and benign pulmonary metastases

**DOI:** 10.1186/1746-1596-5-62

**Published:** 2010-09-22

**Authors:** Ira J Miller, Alan Blank, Suellen M Yin, Allison Mcnickle, Robert Gray, Steven Gitelis

**Affiliations:** 1 Department of Orthopedic Surgery, Rush University Medical Center, 1611 W. Harrison #300 Chicago, IL, 60612, USA; 2 Department of Pathology, Rush University Medical Center, 1611 W. Harrison #300 Chicago, IL, 60612, USA

## Abstract

Giant cell tumor (GCT) of bone is a locally destructive tumor that occurs predominantly in long bones of post-pubertal adolescents and young adults, where it occurs in the epiphysis. The majority are treated by aggressive curettage or resection. Vascular invasion outside the boundary of the tumor can be seen. Metastasis, with identical morphology to the primary tumor, occurs in a few percent of cases, usually to the lung. On occasion GCTs of bone undergo frank malignant transformation to undifferentiated sarcomas. Here we report a case of GCT of bone that at the time of recurrence was found to have undergone malignant transformation. Concurrent metastases were found in the lung, but these were non-transformed GCT.

## Introduction

Giant cell tumor of bone is a locally destructive tumor that occurs predominantly in long bones of post pubertal adolescents and young adults, where it occurs in the epiphysis. The majority are treated by aggressive curettage or resection. Histologically, giant cell tumor of bone classically shows many large multinucleated giant cells with interspersed haphazardly arranged mononuclear cells, and the nuclear features of both elements are described as similar. Some tumors also have areas with a fascicular or storiform pattern devoid of giant cells resembling a benign fibrous histiocytoma. Vascular invasion outside the boundary of the tumor can be seen. The rate of local recurrence varies among centers and is influenced by the completeness of surgical treatment, with high speed burring, adjuvants, and bone cement adding to the effectiveness of curettage treatment[1]. Unresectable tumors such as large sacral masses can be treated with radiation[2]. New therapies targeting the Receptor Activator of NF-κB (RANK) signaling pathway, such as with the anti-RANK ligand antibody denosumomab are in early stages of investigation[3]. Metastasis, with identical morphology to the primary tumor, occurs in a few percent of cases, usually to the lung. These cases are treated with wedge resection with good long term outlook[4]. On occasion giant cell tumors of bone undergo frank malignant transformation to undifferentiated sarcomas. Here we report a case of giant cell tumor of bone that at the time of recurrence was found to have undergone malignant transformation. Concurrent metastases were found in the lung, but these were non-transformed giant cell tumor. Contemporaneous histologically benign pulmonary metastases and locally recurrent giant cell tumor of bone with transformation to sarcoma has not to our knowledge been previously reported.

## Materials and methods

This study was performed with the approval of the Rush University IRB, ORA#: 09092501-CA01. The study included detailed clinical information, imaging and pathology. Tissue was fixed in 10% buffered formalin at room temperature and dehydrated and paraffin embedded in overnight processing. Immunohistochemical stains were performed as follows: The following antibodies were used on Ventana Benchmark and the manufacturer's solution CC1 for antigen retrieval.: CD4 (Biocare Medical, Concord, CA) 1:10 dilution, CD43 (Cell Marque, Rocklin, CA) prediluted, P63 (Fisher Scientific, Pittsburgh, PA) 1:500. Ki-67 immunostaining was performed on the Dako autostainer PLUS with FLEX Envision chemistry using clone MIB-1 (Dako, Carpentaria, CA), at 1:400 dilution after citrate buffer antigen retrieval under pressure in a microwave oven. Images were captured on an Olympus BX41 microscope with a Spot Insight color camera with Spot Advanced software.

## Report of a case

A 29 year-old male was diagnosed with a giant cell tumor of the left proximal tibia at an outside institution in January 2005. He underwent intralesional curettage followed by heat cauterization and methacrylation. Eight months later, in September 2005, the patient had a local recurrence of the tumor which was treated with cement removal and repeat curettage followed by argon beam ablation and repeat cementing.

**Figure 1:**
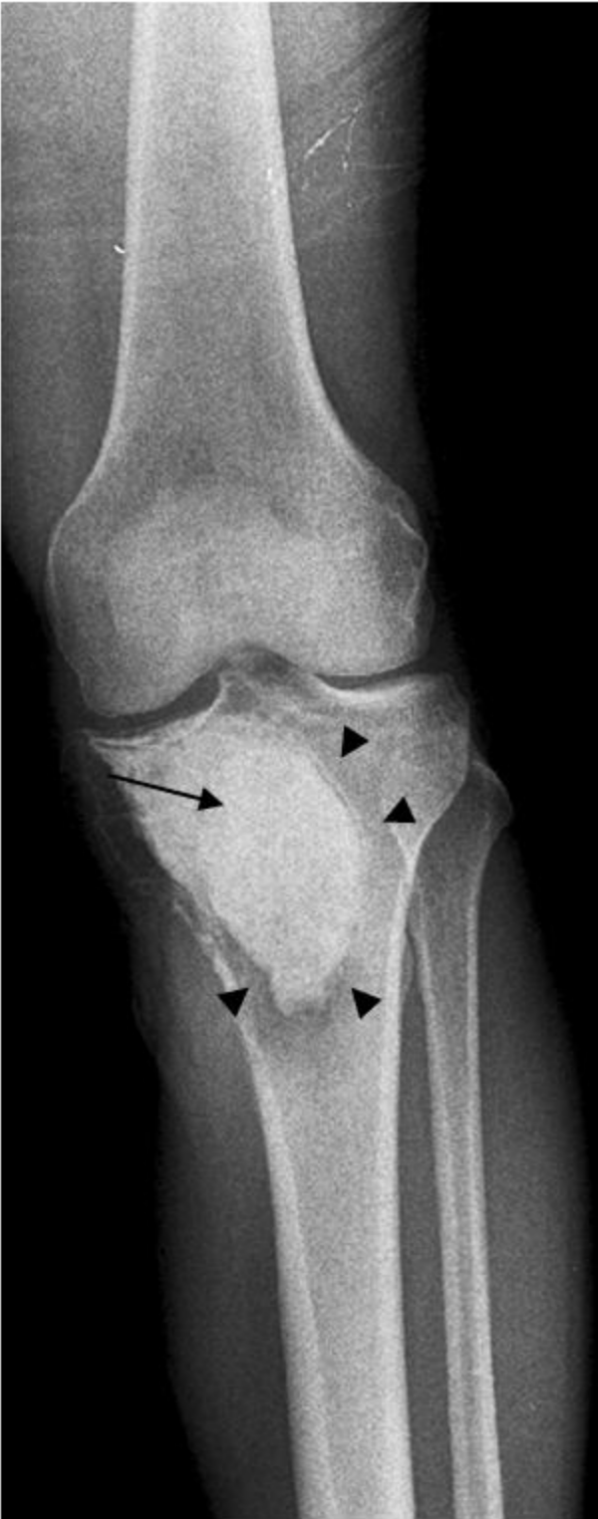
**Radiograph of the recurrent left tibial tumor showing a near circumferential lucency (arrowheads) around the bone cement (arrow)**.

**Figure 2:**
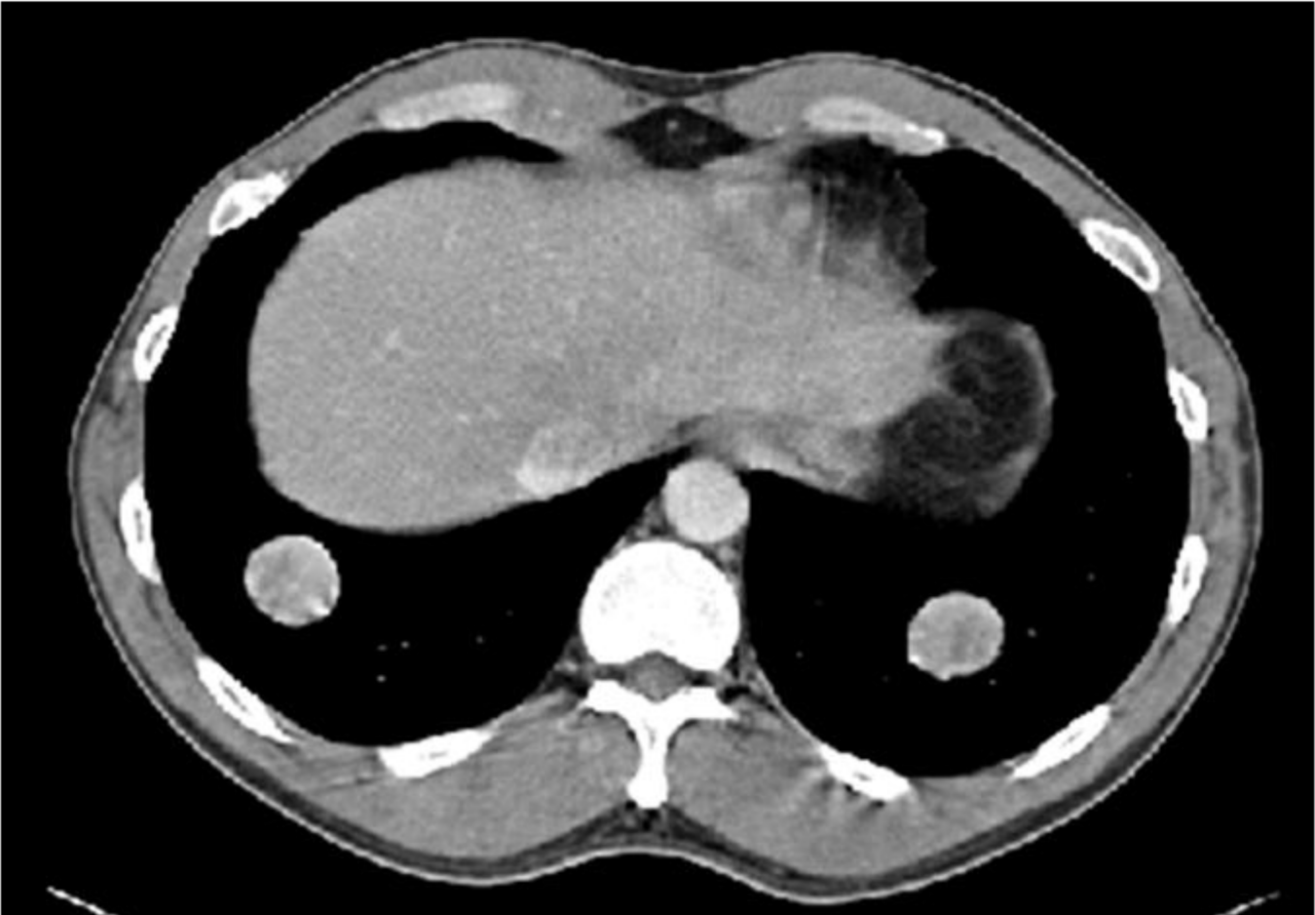
**Chest CT showing bilateral metastatic tumor nodules in the lung bases**.

The patient presented at this institution in June 2009 with severe pain and disability of the left leg at the site of the previous excisions. Radiographs demonstrated a large lucency around the cement (Figure 1). Concurrent staging revealed nodules on chest radiograph, and computerized tomography revealed two nodules in each lung, up to 2.8 cm in dimension (Figure 2). In July 2009, the patient underwent bilateral thoracoscopy-assisted pulmonary resections. Frozen section revealed the pulmonary lesions to be histologically benign. As a result of the benign pulmonary histology, a repeat intralesional excision, cauterization with phenol and methacrylation with internal fixation of the proximal tibia was performed. Intraoperatively, the tumor was recurrent throughout (cephalad, caudad, medial and lateral to the cement) and bulging poster medially to the pes anserine tendons. Fibrous tissue was observed near the patellar ligament insertion. The post-operative course was unremarkable. The permanent paraffin sections confirmed the benign histology in the lung lesions; however, the tibial recurrence was frankly malignant. As a result, this patient received pre-operative chemotherapy given the poor prognosis [5]. Following the chemotherapy, he was taken to surgery, and an en-bloc resection of the upper tibia was performed. He was reconstructed with a modular oncology tibial and knee prosthesis. No residual tumor was seen in the resected specimen. Close follow-up, consisting of chest CT and tibial imaging every 3 months, is ongoing.

## Pathological findings

**Figure 3:**
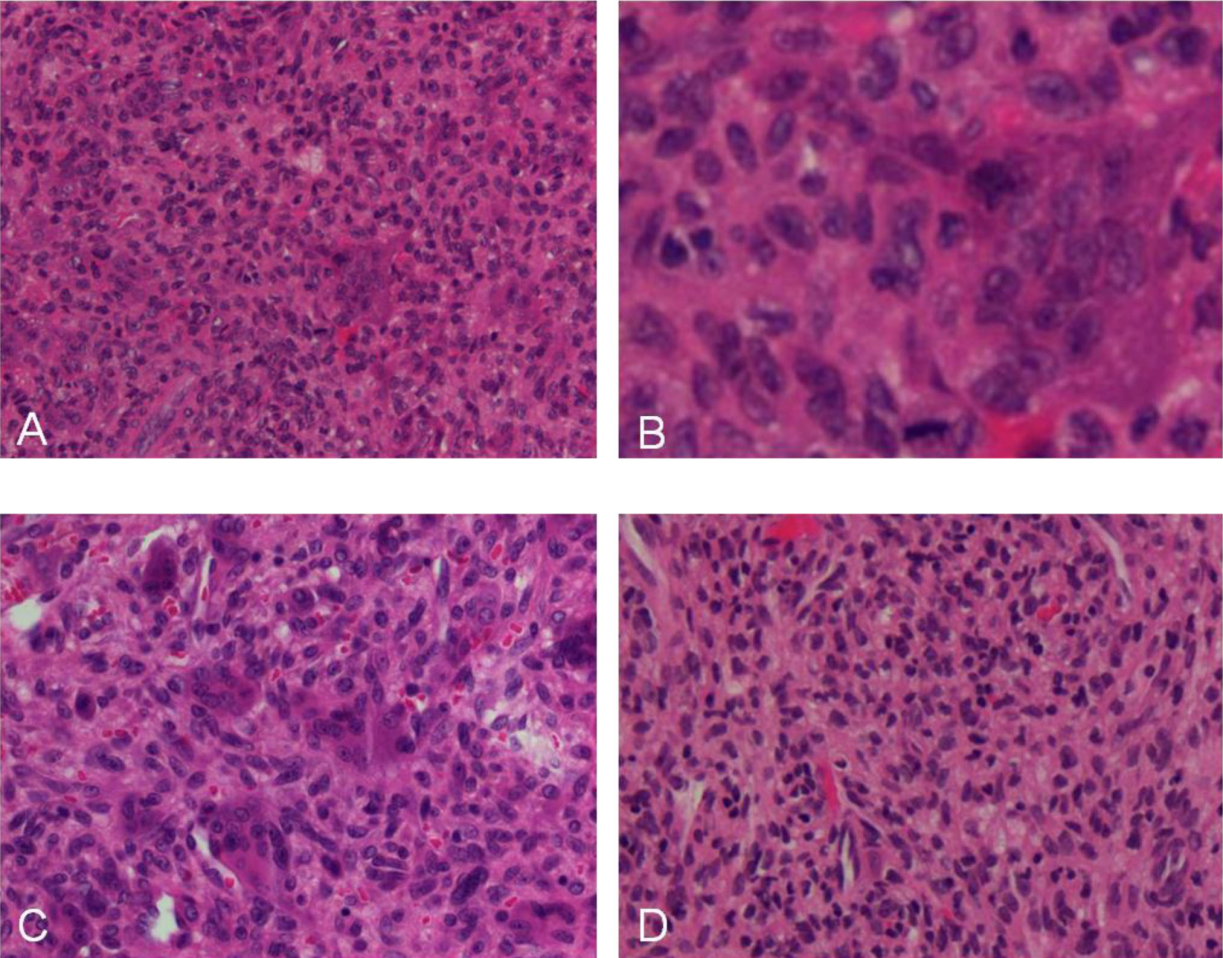
**A, B, C and D**. The tumor at presentation (2005), showing giant cells admixed with round and spindled mononuclear cells (A, original magnification ×230, B, original magnification ×800) and areas with predominantly (C, original magnification ×290) and exclusively (D, original magnification ×250) spindled stromal cells. No atypia was seen.

Histological sections of the tumor from presentation showed classic giant cell tumor of bone with the typical histological patterns (Figure 3). Most areas showed multinucleated giant cells admixed with round and spindled mononuclear cells (A, B). In some areas the spindled stromal component predominated (C), and in one area spindled cells proliferated in the absence of giant cells. No atypia was seen.

**Figure 4:**
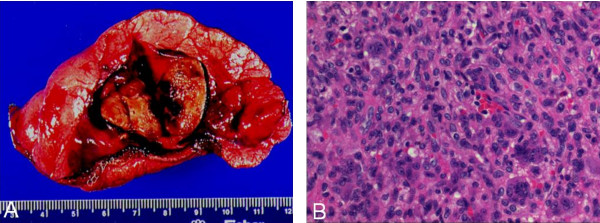
**A and B, Metastatic tumor in a lung wedge resection (2009) grossly showing a well circumscribed mahogany nodule (A), histologically similar to the original tumor (B original magnification ×290)**.

**Figure 5:**
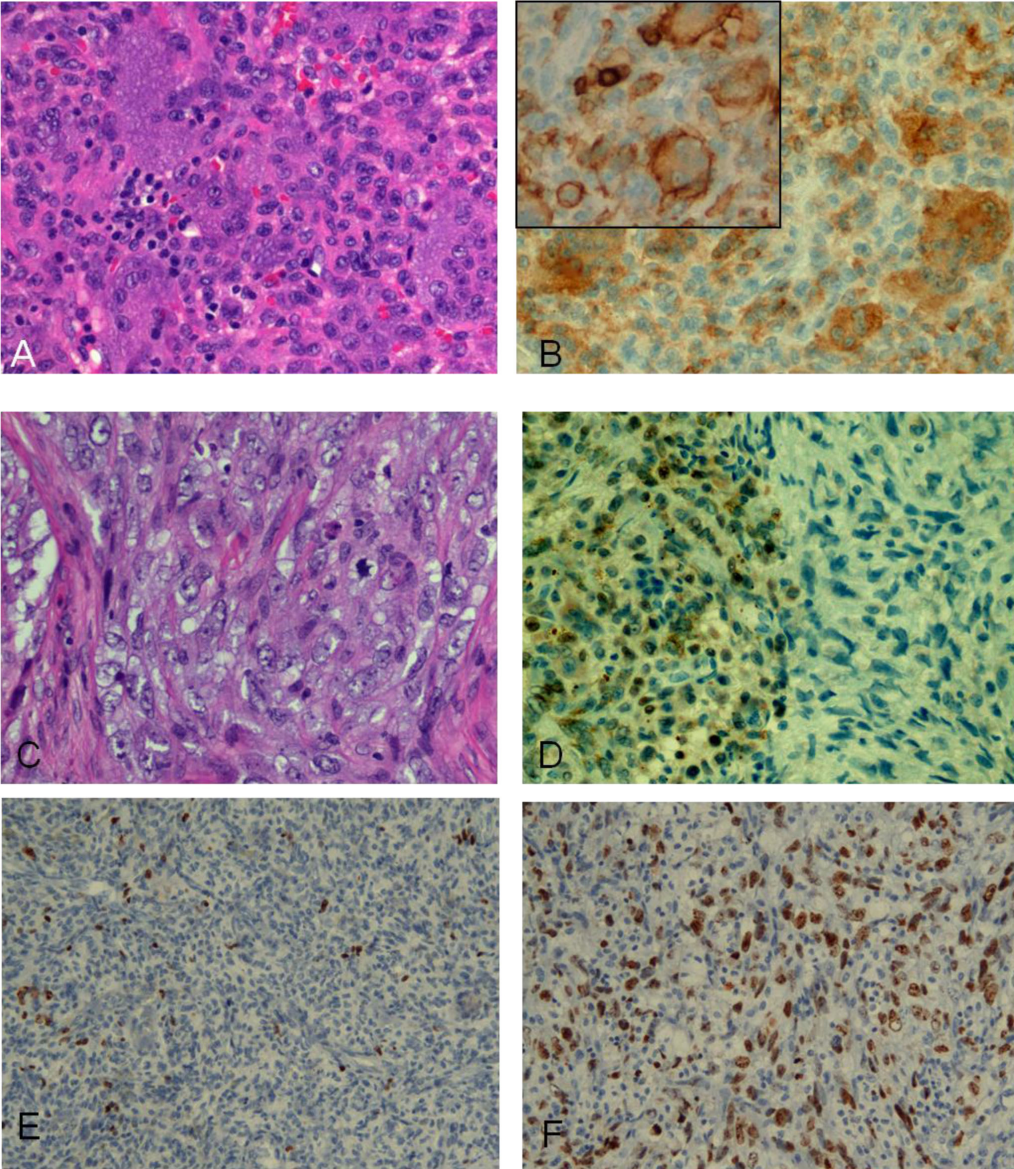
**A, B, C, D, E and F**. Recurrent tibial tumor (2009): area with classic histology of benign giant cell tumor of bone. The nuclei of the two components are similar, with smooth contours and relatively condensed chromatin (A original magnification ×290). Immunostain for CD4 (B original magnification ×290) and CD43 (B, inset; the smaller, darker CD43+ cells are T cells original magnification ×290). Area with frank malignant transformation characterized by large and pleomorphic cells with vesicular and irregularly shaped nuclei, prominent nucleoli and frequent mitotic figures (C). Immunostain for P63 (D, original magnification ×290). Immunostain for MIB-1 in benign (E) and malignant (F) areas (original magnification × 145).

The gross specimen from the tibial excision consisted of fragments of pink-tan and red-brown tissue with pieces of cement and bone fragments. The metastatic tumor nodules resected from the lung (Figure 4) grossly were well circumscribed mahogany nodules and histologically showed essentially identical morphology to the original tumor. Some areas of the recurrent tibial tumor also show classic histology of benign giant cell tumor of bone (Figure 5A). Immunostains distinguish between the monocyte lineage cells--both mononuclear cells and multinucleated giant cells, which are highlighted by the lineage specific markers CD4 and CD43 (Figure 5B, inset; the smaller, darker CD43+ cells are T cells) and the stromal spindle cell component which is negative. About 50% of the tibial tumor showed undifferentiated pleomorphic sarcoma having large spindled cells with vesicular nuclei, prominent nucleoli and scattered mitoses (up to more than 20 per 10 standard high power fields) and abundant apoptotic debris (Figure 5C). The benign component showed nuclear P63 staining in a subset of cells, as has been reported (Figure 5D) [6]. Also, Mib-1 staining is low in the benign component and significantly increased in the malignant component (Figures 5E, F).

## Comment and discussion

Giant cell tumors of bone are neoplasms of stromal cells that recruit a mononuclear population of hematopoietic origin [7]. These monocytes and/or multinucleated giant cells may in turn produce factors that support growth of the stromal cells, but this has not been explored. The onocytederived component is highlighted in immunohistochemical stains for the lysosomal marker CD68 and for monocyte lineage specific markers. The lineage of the stromal cells is still poorly characterized, but a subset variably expresses alkaline phosphatase, similar to osteoblasts [8]. Factors such as monocyte colony stimulating factor (m-CSF) and RANK ligand, which are important for osteoclastogenesis and for fusion of monocytes to form multinucleated giant cells, are expressed in giant cell tumors of bone[9]. Presumably the stromal cells are the source of m-CSF, akin to the situation in localized and diffuse giant cell tumors of tendon sheath/synovium where m-CSF is overexpressed by the neoplastic cells, sometimes secondary to a recurrent translocation[10,11]. Accordingly, cultured giant cell tumor stromal cells are chemoattractant to peripheral blood monocytes[12].

In karyotypic analysis of giant cell tumors of bone, end-to-end fusions of various chromosomes, termed telomeric associations, are seen in most tumors in a subset of cells, and these have been localized by FISH studies to the CD68 negative spindled stromal component[13]. At lower frequency than telomeric associations, clonal chromosome gains, deletions and translocations can also be found in GCT of bone[14]. Translocations occur more frequently in tumors that have more telomeric associations, and they probably result from ensuing problems in separation of dicentric chromosomes during telophase[14]. However, cells with chromosomal abnormalities do not have enough of a growth advantage to dominate the tumor population, and analyses of many metaphases is required to recognize that there are clonal subpopulations in the tumor[14]. In Goronova's study[14] there was no association between karyotype and prognosis. Also, no dominant recurrent cytogenetic abnormality was seen that might give clues to pathogenesis. Thus, this peculiar low level chromosome instability reflects an underlying defect in chromosome maintenance, though probably not due to a generalized defect in telomerase activity[15]. The minor genomic instability seen in vitro is reflected by a low but definite probability of transformation clinically, as seen here. However, because of the current lack of a defined molecular or cytogenetic marker of GCT of bone, formal proof that the rare cases of malignant transformation are due to evolution of the spindle cells of GCT of bone is lacking. An alternative hypothesis would be that that GCT of bone predisposes to malignancy in an unrelated cell--akin to secondary malignancy in the setting of osteonecrosis or Paget's disease of bone. Notably, cases are considered secondary malignant giant cell tumors even if, on recurrence, only the malignant component is seen.

This case illustrates a sarcoma arising at the site of a locally recurrent GCT of bone, with concurrent non-sarcomatous metastases. Local recurrence of GCT of bone is common, occurring 10-20% of the time[16-18], and depends on the aggressiveness of the initial surgery[19]. Metastasis with "benign" morphology, most commonly to the lung, was reported in an average of 3% of cases (table 1), and more than half occurred in patients who also had local recurrence.

**Table 1:** Reported incidence of "benign" metastasis in giant cell tumor of bone.

	*# Cases (total)*	*Metastatic (%)*	*Reference#*
	214	7	[1]
	624	14 (2.2)	[4]
	470	21 (4.5)	[26]
**Total**	1308	42 (3.2)	

Diagnosis of transformation relies on overt malignant cytological features, as necrosis and scattered mitoses can be seen in the usual "benign" giant cell tumor of bone. Immunohistochemical studies are not required for the diagnosis of transformation. Usually there is no evident lineage of maturation, but cases of osteosarcoma have been reported[9,20-24]. The incidence of malignancy in GCT varies considerably among studies, with an overall rate of approximately 5% considering the largest series together (table 2). About half were associated with prior irradiation (table 2), but transformation was found in the initial resection in about one third of cases (table 2). Secondary transformation had a latency of up to 28 years[20] in a case that without radiotherapy. Given the wide range of reported incidence of malignancy in giant cell tumor of bone among studies, it is probable that this entity has been over diagnosed in the reported series. Of note, in the publications with the highest rates of malignancy, there was no pathological slide review. In Bertoni's study, pathological review was performed[20], and as a result, the initial diagnosis was revised to something other than malignant transformation of giant cell tumor in 12 of 26 cases initially considered for which slides were available. Anract's study, which did not include pathological review, includes 5 cases with "well differentiated fibrosarcoma"[5], which is much different from the more widely recognized high grade transformation. Likewise, in Domovitov's report, 57% of the cases had the curious diagnosis of "focally" malignant histology. Accordingly, the outcomes in their study were optimistic, with 80% of patients predicted to be recurrence free at 5 years based on their data, compared to the 10-50% overall survival estimated in most other series. Thus, excluding radiation-induced transformation cases, and considering only series with pathological review, the incidence of malignant transformation of giant cell tumor of bone is only about 1%, which is the rate cited in the WHO publication[25].

**Table 2:** Reported incidence of malignancy associated with giant cell tumor of bone in large published series

	*# Cases (total)*	*Total Malig (%)*	*1° (%)*	*2° (%)*	*Post radiation*	*Reference#*
	193	29 (15)	17 (9)	9 (5)	3 (2)	[5]
	924	17 (2)	5 (0.5)	6 (0.5)	6 (0.5)	[20]
	627	31 (5)	?	?	25 (4)	[27] includes cases cited in [21]
	195	17 (9)	3 (1.5)	2 (1)	12 (6)	[28]
	607	13 (2)	6 (1)	7 (1)	?	[29]
	275	26 (10)	?	?	5 (2)	[30]
**Total**	2821	133 (4.7)				

## Conclusion

We report here the case of a giant cell tumor of the proximal tibia of a 29 year old man treated with aggressive curettage that recurred 4 years later with malignant transformation and "benign" metastases to the lung. Recurrence, malignant transformation, and metastasis with "benign" morphology all occur in giant cell tumor of bone. This is the first report of all three occurring in a single patient. The influence of local recurrence on malignant transformation and pulmonary metastases is largely unknown. Malignant transformation has been reported most frequently with radiation even in nonrecurrent tumors. Similarly, benign pulmonary metastases have been reported to occur in recurrent and nonrecurrent tumors. The current report adds little to the discussion of the fate of locally recurrent giant cell tumor of bone. The molecular pathways leading to giant cell tumors of bone are still largely uncharacterized.

## Competing interests

The authors declare that they have no competing interests.

## Authors' contributions

IM reviewed the pathology and wrote the discussion of the molecular biology. AB formatted the article. SY and AM reviewed the pertinent literature. RG obtained the approval of the institutional review board and patient's consent. SG prepared the clinical case report. All authors read and approved the final manuscript.

## Consent

Written informed consent was obtained from the patient for publication of this case report and accompanying images. A copy of the written consent is available for review by the Editor-in Chief of this Journal.

## References

[B1] Balke M Schremper L Gebert C Ahrens H Streitbuerger A Koehler G Hardes J Gosheger G Giant cell tumor of bone: treatment and outcome of 214 cases J Cancer Res Clin Oncol 2008 134 9 969 78 10.1007/s00432-008-0370-x 18322700 PMC12160765

[B2] Caudell JJ Ballo MT Zagars GK Lewis VO Weber KL Lin PP Marco RA El-Naggar AK Benjamin RS Yasko AW Radiotherapy in the management of giant cell tumor of bone Int J Radiat Oncol Biol Phys 2003 57 1 158 65 12909228 10.1016/s0360-3016(03)00416-4

[B3] Thomas DM Skubitz KM Giant cell tumour of bone Curr Opin Oncol 2009 21 4 338 44 10.1097/CCO.0b013e32832c951d 19444102

[B4] Dominkus M Ruggieri P Bertoni F Briccoli A Picci P Rocca M Mercuri M Histologically verified lung metastases in benign giant cell tumours--14 cases from a single institution Int Orthop 2006 30 6 499 504 10.1007/s00264-006-0204-x 16909252 PMC3172731

[B5] Anract P De Pinieux G Cottias P Pouillart P Forest M Tomeno B Malignant giant-cell tumours of bone. Clinico-pathological types and prognosis: a review of 29 cases Int Orthop 1998 22 1 19 26 10.1007/s002640050201 9549577 PMC3619644

[B6] Dickson BC Li SQ Wunder JS Ferguson PC Eslami B Werier JA Turcotte RE Kandel RA Giant cell tumor of bone express p63 Mod Pathol 2008 21 4 369 75 10.1038/modpathol.2008.29 18311114

[B7] Werner M Giant cell tumour of bone: morphological, biological and histogenetical aspects Int Orthop 2006 30 6 484 9 10.1007/s00264-006-0215-7 17013643 PMC3172738

[B8] Morgan T Atkins GJ Trivett MK Johnson SA Kansara M Schlicht SL Slavin JL Simmons P Dickinson I Powell G Choong PF Holloway AJ Thomas DM Molecular profiling of giant cell tumor of bone and the osteoclastic localization of ligand for receptor activator of nuclear factor kappaB Am J Pathol 2005 167 1 117 28 15972958 10.1016/s0002-9440(10)62959-8 PMC1603441

[B9] Atkins GJ Haynes DR Graves SE Evdokiou A Hay S Bouralexis S Findlay DM Expression of osteoclast differentiation signals by stromal elements of giant cell tumors J Bone Miner Res 2000 15 4 640 9 10.1359/jbmr.2000.15.4.640 10780856

[B10] Cupp JS Miller MA Montgomery KD Nielsen TO O'Connell JX Huntsman D van de Rijn M Gilks CB West RB Translocation and expression of CSF1 in pigmented villonodular syn vitis, tenosynovial giant cell tumor, rheumatoid arthritis and other reactive synovitides Am J Surg Pathol 2007 31 6 970 6 10.1097/PAS.0b013e31802b86f8 17527089

[B11] West RB Rubin BP Miller MA Subramanian S Kaygusuz G Montgomery K Zhu S Marinelli RJ De Luca A Downs-Kelly E Goldblum JR Corless CL Brown PO Gilks CB Nielsen TO Huntsman D van de Rijn M A landscape effect in tenosynovial giant-cell tumor from activation of CSF1 expression by a translocation in a minority of tumor cells Proc Natl Acad Sci USA 2006 103 3 690 5 10.1073/pnas.0507321103 16407111 PMC1325107

[B12] Salerno M Avnet S Alberghini M Giunti A Baldini N Histogenetic characterization of giant cell tumor of bone Clin Orthop Relat Res 2008 466 9 2081 91 10.1007/s11999-008-0327-z 18543051 PMC2492994

[B13] Moskovszky L Szuhai K Krenács T Hogendoorn PC Szendroi M Benassi MS Kopper L Füle T Sápi Z Genomic instability in giant cell tumor of bone. A study of 52 cases using DNA ploidy, relocalization FISH, and array-CGH analysis Genes Chromosomes Cancer 2009 48 6 468 79 10.1002/gcc.20656 19242928

[B14] Gorunova L Vult von Steyern F Storlazzi CT Bjerkehagen B Follerås G Heim S Mandahl N Mertens F Cytogenetic analysis of 101 giant cell tumors of bone: nonrandom patterns of telomeric associations and other structural aberrations Genes Chromosomes Cancer 2009 48 7 583 602 10.1002/gcc.20667 19396867

[B15] Forsyth RG De Boeck G Bekaert S De Meyer T Taminiau AH Uyttendaele D Roels H Praet MM Hogendoorn PC Telomere biology in giant cell tumour of bone J Pathol 2008 214 5 555 63 10.1002/path.2301 18278785

[B16] Turcotte RE Wunder JS Isler MH Bell RS Schachar N Masri BA Moreau G Davis AM Giant cell tumor of long bone: a Canadian Sarcoma Group study Clin Orthop Relat Res 2002 397 248 58 10.1097/00003086-200204000-00029 11953616

[B17] Vult von Steyern F Bauer HC Trovik C Kivioja A Bergh P Holmberg Jörgensen P Follerås G Rydholm A Treatment of local recurrences of giant cell tumour in long bones after curettage and cementing. A Scandinavian Sarcoma Group study J Bone Joint Surg Br 2006 88 4 531 5 10.1302/0301-620X.88B4.17407 16567792

[B18] Zhen W Yaotian H Songjian L Ge L Qingliang W Giant-cell tumour of bone. The long-term results of treatment by curettage and bone graft J Bone Joint Surg Br 2004 86 2 212 6 10.1302/0301-620X.86B2.14362 15046435

[B19] Su YP Chen WM Chen TH Giant-cell tumors of bone: an analysis of 87 cases Int Orthop 2004 28 4 239 43 10.1007/s00264-004-0564-z 15160253 PMC3456930

[B20] Bertoni F Bacchini P Staals EL Malignancy in giant cell tumor of bone Cancer 2003 97 10 2520 9 10.1002/cncr.11359 12733152

[B21] Rock MG Sim FH Unni KK Witrak GA Frassica FJ Schray MF Beabout JW Dahlin DC Secondary malignant giant-cell tumor of bone. Clinicopathological assessment of nineteen patients J Bone Joint Surg Am 1986 68 7 1073 9 3745247

[B22] Brien EW Mirra JM Kessler S Suen M Ho JK Yang WT Benign giant cell tumor of bone with osteosarcomatous transformation ("dedifferentiated" primary malignant GCT): report of two cases Skeletal Radiol 1997 26 4 246 55 10.1007/s002560050230 9151375

[B23] Hoch B Inwards C Sundaram M Rosenberg AE Multicentric giant cell tumor of bone. Clinicopathologic analysis of thirty cases J Bone Joint Surg Am 2006 88 9 1998 2008 10.2106/JBJS.E.01111 16951117

[B24] Marui T Yamamoto T Yoshihara H Kurosaka M Mizuno K Akamatsu T De novo malignant transformation of giant cell tumor of bone Skeletal Radiol 2001 30 2 104 8 10.1007/s002560000305 11310196

[B25] Fletcher CDM Unni KK Mertens F (eds.) World Health Organization Classification of Tumours. Pathology and Genetics of Tumours of Sorf Tissue and Bone 2002 IARC Press, Lyon

[B26] Viswanathan S Jambhekar NA Metastatic giant cell tumor of bone: are there associated factors and best treatment modalities? Clin Orthop Relat Res 2010 468 3 827 33 10.1007/s11999-009-0966-8 19597900 PMC2816751

[B27] Unni KK Inwards CY Bridge JA Kindblom LG Wold LE (eds) Tumors of the bones and joints, 4th series ed 2005 AFIP Atlas of Tumor Pathology. American Registry of Pathology, Washington, DC

[B28] Dahlin DC Cupps RE Johnson EW Jr Giant-cell tumor: a study of 195 cases Cancer 1970 25 5 1061 70 10.1002/1097-0142(197005)25:5<1061::AID-CNCR2820250509>3.0.CO;2-E 4910256

[B29] Gong LH Sun XQ Meng SQ Huang XY [Giant cell tumor of bone and malignancies in giant cell tumor: a clinicopathologic analysis] Zhonghua Bing Li Xue Za Zhi 2009 38 5 312 5 19575873

[B30] Domovitov SV Healey JH Primary Malignant Giant-Cell Tumor of Bone Has High Survival Rate Ann Surg Oncol 2010 17 3 694 701 10.1245/s10434-009-0803-z 19902306

